# Targeting the ‘garbage-bin’ to fight cancer: HDAC6 inhibitor WT161 has an anti-tumor effect on osteosarcoma and synergistically interacts with 5-FU

**DOI:** 10.1042/BSR20210952

**Published:** 2021-08-06

**Authors:** Consolato M. Sergi

**Affiliations:** 1 AP Division/Pathology Laboratories, Children’s Hospital of Eastern Ontario, University of Ottawa, 401 Smyth Rd, Ottawa, Ontario K1H 8L1, Canada; 2 Department of Orthopedics, Tianyou Hospital, Wuhan University of Science and Technology, Wuhan, Hubei, China

**Keywords:** 5-FU, apoptosis, osteosarcoma, PTEN, synergistic inhibition, WT161

## Abstract

An imbalance between protein aggregation and protein degradation may induce ‘stress’ in the functionality of the endoplasmic reticulum (ER). There are quality control (QC) mechanisms to minimize misfolding and to eliminate misfolded proteins before aggregation becomes lethal for the cell. Proper protein folding and maturation is one of the crucial functions of the ER. Chaperones of the ER and folding enzymes guarantee correct conformational maturation of emerging secretory proteins. Histone deacetylase (HDAC) 6 (HDAC6) is a masterpiece coordinating the cell response to protein aggregate formation. The balance between HDAC6 and its partner Valosin-containing protein/p97 determines the fate of polyubiquitinated misfolded proteins. WT161 is a terrific, selective, and bioavailable HDAC6 inhibitor. WT161 selectively inhibits HDAC6 and adequately increases levels of acetylated α-tubulin. This compound induces accumulation of acetylated tubulin and cytotoxicity in multiple myeloma (MM) cells. In this journal, Sun et al. (*Biosci. Rep.* **41**, DOI: 10.1042/BSR20203905) identified that WT161 suppresses the cell growth of osteosarcoma cells. This discovery opens the door to future chemotherapeutic regimens of this bone neoplasm.

The synthesis of proteins is not a smooth road, but each protein undergoes a series of pitfalls to become fully functional. Protein folding is a natural event and often occurs in cells, particularly for relatively small proteins. A sequence of folding intermediates takes place, and during this folding, the exposure of hydrophobic domains leads to incongruous associations, which consequently trigger protein aggregation. The balance between unfolded polypeptides, misfolded, and folded macromolecules determine the viability of the cell or, in other terms, a state of toxicity if such a setting is unbalanced. Ultimately, a protein should either correct its folding or aggregate. The fate is determined by genetic and environmental events, including mutations, modification, translation misreading, or, even, unequal synthesis of subunits. Also, pH, temperature, ionic strength, and redox environment substantially influence cellular misfolding. Thus, nature has two mechanisms to deal with protein misfolding.

Accumulation of misfolded proteins can induce disease, and some of these, are known in the medical literature as amyloid diseases. Alzheimer’s disease is the most prevalent amyloid disease affecting approx. 10 percent of the adult population aged 65 years or over living in the United States and Canada [1,2]. Other neurodegenerative diseases associated with the accumulation of misfolded proteins are Parkinson’s disease and Huntington’s disease. No matter which type, the risk of contracting any of these diseases increases tremendously with age [3]. The senescence is indeed associated with a perturbation of the synthesis, folding, and degradation of proteins.

Consequently, the production and accumulation of misfolded proteins that form aggregates ensues. Non-neurodegenerative diseases associated with the collection of misfolded proteins are also common worldwide. These conditions are type 2 diabetes mellitus, atherosclerosis, cataract, and hemodialysis-related disorders, among others. In all these diseases, there is the expression of a protein outside its usual context. This situation leads to an irreversible change of the protein, which seems to be connotated as a ‘sticky’ configuration because it is rich in β sheets. Accordingly, the protein molecules ‘stick’ with each other. Protein aggregation may also be a consequence of protein hyperphosphorylation, self-catalytic conformational conversion, or genetic mutations leading to an unstable protein [3–8].

These are quality control (QC) mechanisms to minimize misfolding and to eliminate misfolded proteins before aggregation becomes lethal for the cell. Proper protein folding and maturation is one of the crucial functions of the endoplasmic reticulum (ER). ER chaperones and folding enzymes guarantee correct conformational maturation of emerging secretory proteins. The ER protein QC system closely screens protein folding using specific factors that support newly synthesized proteins to accomplish their final conformation. ER chaperones are critical to many aspects of this crucial ER function, whether in protein folding modes, as calcium binders, as sensors of stress such as the unfolded protein response, or due to cell-surface localization or extracellular release, as immune modulators [9]. Furthermore, some scarcity of key components can determine imbalances between protein aggregation and degradation in the proteasome, which remains crucial as the cellular machinery involved in the degradation of aging proteins and autophagy inhibition [10]. The severity of degenerative diseases correlates with increased mitochondrial dysfunction, oxidative stress, cytoplasmic membrane permeability, and abnormal ion (e.g., calcium) concentration [11,12].

In the cell, the ER plays a crucial role in the biogenesis of secretory and cell surface proteins. The recently synthesized polypeptides enter the ER using a proteinaceous pore called the translocon [13–19]. In addition, there are ER-resident enzymes, which guide protein folding towards the native state by post-translational modification and chaperoning. Proteins that fail to incorporate their native conformation remain in the ER and are eventually targeted for ER-associated degradation (ERAD). Protein homeostasis or proteostasis includes processes governing protein production, QC, and degradation. Efficiency is vital, and efficient protein folding within the ER necessitates tight matching of a load of synthesized proteins entering the organelle with the capacity of its folding apparatus. Suppose the burden of client proteins outweighs the ability. In that case, the cell experiences ‘ER stress,’ representing a potential life-threatening status because there is an accumulation of unfolded aggregation-prone species. In this setting, an unfolded protein response (UPR) is employed to restore proteostasis [19,20].

Four classes of agents can induce ER stress [8,15,21]. These classes are hypoxia, inhibitors of glycosylation, calcium metabolism, and reducing agents. In addition, the ER stress response can result in the activation of apoptosis, which is regulated in mammalians by PERK, which is a transmembrane kinase, ATF6, which is a transmembrane transcription factor, and IRE1, a transmembrane RNase. Tumor cells are exposed to several environmental stressors, including hypoxia, limited nutrients, acidosis, and chemotherapy, but some activation of the stress response could confer resistance to drugs.

Cancer drug resistance is a complex phenomenon. It is influenced by numerous phenomena or processes, including drug inactivation, drug target alteration, DNA damage repair, drug efflux, cell death inhibition, epithelial–mesenchymal transition, inherent cell heterogeneity, epigenetic effects, as well as any combination of these mechanisms [22]. Currently, combination therapy is the best treatment option because it often prevents the development of drug resistance. Such treatment regimens are often considered and developed to act against the increasing prevalence of drug resistance in cancers. At the origin, cancer progenitor cells are often drug resistant and the persistent progenitor cells can remain stationary or migrate to other sites during metastasis [23].

Molecular chaperones have progressed to assist with folding newly synthesized proteins and refolding proteins damaged by stress and cellular injury. An essential survival mechanism is a ubiquitin–proteasome system, which is the first QC mechanism and degrades proteins that cannot fold correctly. The proteasome is a multisubunit complex localized in the cytosol and nucleus. It can degrade cytosolic, nuclear, secretory, and transmembrane proteins into smaller peptides. Misfolded proteins are retained in the lumen or membrane of the ER and subsequently retrotranslocated back to the cytosol and transported to the proteasome.

There is full acknowledgment in science that a disorder in protein regulation can be highly deleterious for the organism. A large portion of molecular interactions depend on the complementary interaction between structurally organized proteins and intrinsically disordered proteins (IDPs), i.e., proteins that lack a fixed or ordered three-dimensional structure. The disorder is often identified in intrinsically disordered regions (IDRs) within a well-structured protein. Thus, IDPs include proteins that contain IDRs as well as fully disordered proteins. The structural condition confers various advantages to the proteins, including rapid and specific binding and carrying out some interdependent functions. A disordered state occurs in proteins involved with transcription, signaling, phosphorylation, RNA processing, cytoskeletal organization, ion transport, ubiquitination, cell cycle control, as well as other highly regulated biological mechanisms. However, considering an evolutionary point of view, intrinsic disorders in proteins might have been the stimulus behind many of the adaptability processes found in proteins and organisms. The process called ‘ERAD’ targets misfolded proteins, which can be retained in the ER and retrotranslocated into the cytosol for proteasomal degradation. As indicated above, ubiquitination is one mechanism involved in a disordered state of a protein and a form of a post-translational modification. In detail, ubiquitination is the biochemical process in which proteins are targeted by ubiquitin, a polypeptide constituted by 76 amino acids. Ubiquitination, which occurs intracellularly in eukaryotes, is temporally controlled. It is a highly coordinated process that plays significant roles in various pathways during cell life, death, and health and disease [24]. The ubiquitination of a protein often results in protein degradation via the ubiquitin–proteasome pathway, although it can also alter protein–protein interaction. Ubiquitination is a three-step process involving three enzymes: ubiquitin-activating enzyme (E1), ubiquitin-conjugating enzyme (E2), and ubiquitin-protein ligase (E3). These reactions promote the subsequent linkage of ubiquitin molecules to a protein attached to any of the seven lysine amino acids or the N-terminus of the ubiquitin molecule. Ubiquitin chains are added, resulting in polyubiquitination. This process leads to the initiation of proteolysis of the substrate by serving as the recognition signal for the 26S proteasome.

In case of complex or impossible folding, degradation does not occur promptly. Thus, the interaction of proteins with other unfolded or partially folded proteins leads to the formation of aggregates. At this point, the aggresome pathway, the second QC mechanism, plays a significant role. This proteasome-independent pathway eliminates misfolded polyubiquitinated proteins, known as the aggresome. The aggresome pathway is then used by the cells to destroy protein aggregates. Aggresomal particles that arise from nascent chains seem to be at the start of the initial aggregation process. They are transferred toward the microtubule organizing center (MTOC) (Figure 1). At this localization, the particles are sequestered into a single large cellular garbage bin-like structure, which is indeed known as the aggresome [25,26]. The transfer of the particles toward the MTOC is an active process requiring energy, well-functioning mitochondria, intact microtubules, and an integer motor dynein–dynactin engine, which is also important for cilia movements [27–29]. Transmission electron microscopy of aggresomes identifies multiple loosely associated particles. However, aggresomes are not just ‘garbage’ bins, but recycling departments able to recruit cytosolic components, including chaperones, ubiquitination enzymatic armamentarium and proteasome subunits, to facilitate the clearance of aggregated proteins. Some of these chaperones are particularly essential, including heat shock protein (HSP) 40 and HSP70. The clearance of aggregated proteins seems to activate autophagy, which is involved in the degradation of various structures ranging from small structures and large organelles such as peroxisomes and mitochondria [10]. The aggresome and autophagy pathways are linked. The parkin-mediated K63-linked polyubiquitination appears to couple misfolded proteins to the dynein motor. It occurs through the interaction with histone deacetylase 6 (HDAC6).

**Figure 1:**
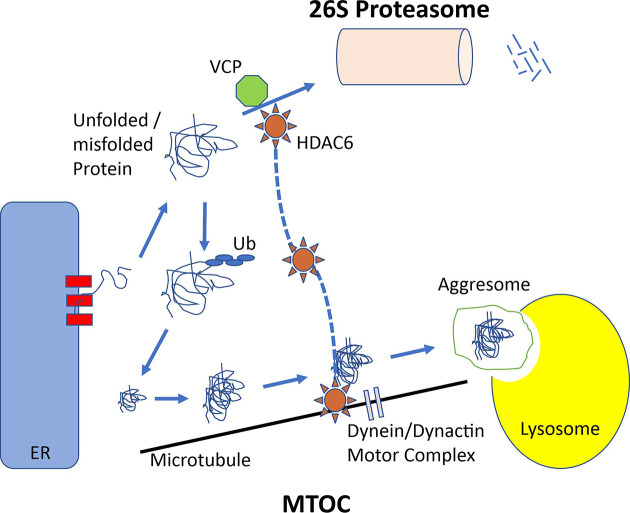
Unfolded or misfolded proteins can originate from proteins damaged by stress, from translating polysomes inappropriately, from proteins retrotranslocated or from the ER to the cytosol These unfolded/misfolded proteins, which fail to fold correctly, may be degraded by the proteasome. Alternatively, they can form aggregates throughout the cells. These aggregates are transported in a microtubule-dependent manner to the MTOC. This apparatus requires the dynein/dynactin motor complex. HDAC6 acetylates α-tubulin. Also, it associates with dynein to facilitate transport of aggregated macromolecules through the cytosol to lysosomes for degradation. HDAC6 is key in coordinating the cell response to protein aggregate formation. Balance between HDAC6 and its partner VCP/p97 ultimately assigns the fate of polyubiquitinated misfolded proteins. The recruitment of HDAC6 to ubiquitinated proteins leads to the induction of HSP90-dependent pathway that stimulates protection against cell stress.

Consequently, aggresomes are formed, and clearance by autophagy occurs. HDAC6 acetylates α-tubulin and associates it with dynein. This process facilitates the cytosolic transport of ‘aggregated’ macromolecules to the lysosomal compartment for degradation. Tyrosination of α-tubulin controls the initiation of dynein–dynactin motility [30]. Thus, HDAC6 is a masterpiece coordinating the cell response to protein aggregate formation. The balance between HDAC6 and its partner Valosin-containing protein (VCP)/p97 determines the fate of polyubiquitinated misfolded proteins. Nonselective inhibition of HDAC6 with vorinostat (suberoylanilide hydroxamic acid, SAHA) or panobinostat (LBH589) blocks lysosomal protein degradation. However, the side-effect profile of the pan-HDAC inhibitors includes fatigue, diarrhea and thrombocytopenia, and this spectrum may limit the clinical utility of this treatment. Thus, the selective targeting HDAC6 could be a potential approach to overcome drug resistance in cancer while reducing the overall toxicity seen using less selective HDAC inhibitors.

Osteosarcoma is the most common primary bone tumor in children, adolescents, and young adults and comprises approx. 20% of primary bone sarcomas. The pediatric age group is most concerned as the peak incidence of the osteosarcoma tumor coincides with rapid skeletal bone growth. Osteosarcoma is slightly more prevalent in males, with most cases (60–70%) affecting the 15–25 age group. Although the incidence of osteosarcoma is lower than other malignancies, this tumor is characterized by a high mortality rate and early distance metastasis, especially to the lungs. Management of osteosarcoma consists of surgery, along with radiotherapy and chemotherapy. The current chemotherapy regimen consists of cisplatin, doxorubicin, methotrexate, and ifosfamide. Despite advancements in medical treatment, the overall survival rate for osteosarcoma, especially in patients with metastasis, is still low. Patients diagnosed with localized disease harbor a 5-year survival rate of approx. 67% (two-thirds), compared with patients with metastasis, whose 5-year survival rate is 20–30% [31,32]. The axis constituted by the insulin-like growth factor (IGF) and the IGF-binding protein (IGFBP) influences the proliferation and survival of various tumors, including osteosarcoma, a malignant bone cancer typically diagnosed in adolescents and young adults. IGFBP-3 is a component of a protein family that can bind IGF-I. Thereby, it regulates the mitogenic activity of IGF-I in the extracellular environment. The tumor suppressor p53 transcriptionally activates the IGFBP-3 gene, and it is assumed that increased expression of IGFBP-3 contributes to p53-dependent apoptosis [31,33–37].

WT161 is a terrific, selective, and bioavailable HDAC6 inhibitor whose chemical formula is depicted in Figure 2. WT161 selectively inhibits HDAC6 and adequately increases levels of acetylated α-tubulin. This compound induces accumulation of acetylated tubulin and cytotoxicity in multiple myeloma (MM) cells [38]. Although WT161 as a single agent does not cause ER stress, it mediated hyperacetylation and inhibition of HSP90 chaperone function, increasing the intracellular levels of polyubiquitinated proteins *in vitro* combined with other treatments [38,39]. WT161 triggers apoptotic cell death in several cell lines, including MCF7, T47D, BT474, and MDA-MB231 cells, which are associated with decreased EGFR, HER2, and ERα and downstream signaling. *In vivo* experiments have identified that WT161 significantly inhibits *in vivo* MCF7 cell growth, associated with down-regulation of ERα, in a murine xenograft model [39].

**Figure 2:**
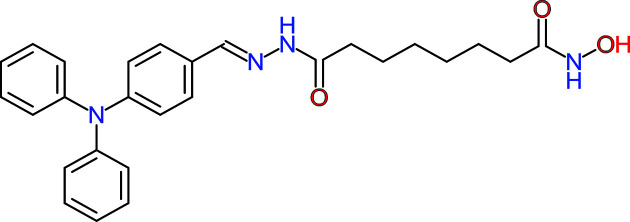
Chemical formula of WT161 The compound, whose the formal chemical name is 8-(hydroxyamino)-8-oxo-octanoicacid,2-[[4-(diphenylamino)phenyl]methylene] hydrazide and the molecular formula is C_27_H_30_N_4_O_3_, belongs to the class of hydrazides. Hydrazides in organic chemistry are a class of organic compounds with the formula R-NHNH_2_ where R is acyl, sulfonyl, or phosphoryl.

Sun et al. identified that WT161 suppresses the cell growth of osteosarcoma cells [40]. These authors treated osteosarcoma cell lines (U2OS and MG63) with WT161. The enhancement of acetyl-α-tubulin concentration upon WT161 treatment showed the efficiency of WT161. Also, a viability assay showed that WT161 suppressed the growth of malignant cells in a dose- and time-dependent manner. The authors also consolidated their results by applying a colony formation assay, which showed that WT161 inhibits the colony formation of osteosarcoma cells in a dose-dependent way. Also, WT161 induced the apoptosis of osteosarcoma cells demonstrated by flow cytometry and the measurement of cleaved PARP. WT161 increased the apoptosis of osteosarcoma cells mainly through regulating PTEN/protein kinase-B signaling pathway. Silencing of PTEN reduced WT161-induced cell apoptosis of tumor cells. Thus, WT161 increases the apoptosis of osteosarcoma cells mainly through the PTEN/AKT signaling pathway regulation.

Moreover, WT161 shows synergistic effects on osteosarcoma cells combined with 5-FU in a mouse xenograft model. WT161 and 5-FU inhibited the growth and weight of osteosarcoma. Previously, the treatment of osteosarcoma with 5-azacytidine significantly increased the expression of PTEN and down-regulated AKT signaling. This process caused apoptosis of osteosarcoma cells [28]. Dr. Sun et al. found that WT161 increased PTEN expression and inhibited the downstream PI3K/Akt signal pathway, thus inducing apoptosis of osteosarcoma cells. The up-regulation of PTEN or inactivation of its downstream signal PI3K/AKT pathway is crucial. It increases the number of dead cells following 5-FU administration on cancer cells. Moreover, it seems to overcome drug resistance, which is a significant burden for oncology. These results are spectacularly promising. They would suggest that WT161 and 5-FU may have a synergistic effect on osteosarcoma *in vivo*. These findings provide evidence that WT161 might become a promising agent against osteosarcoma in the nearest future.

## Abbreviations

ATF6: activating transcription factor 6
ER: endoplasmic reticulum
ERAD: ER-associated degradation
FU/5FU: Fluorouracil / 5-Fluorouracil (C_4_H_3_FN_2_O_2_)
HDAC: histone deacetylase
HSP: heat shock protein
IDP: intrinsically disordered protein
IDR: intrinsically disordered region
IGF: insulin-like growth factor
IGFBP: IGF-binding protein
IRE1: inositol-requiring enzyme 1
MTOC: microtubule organizing center
PERK: protein kinase RNA-like endoplasmic reticulum kinase
QC: quality control
